# Changes in Brain Structure, Function, and Network Properties in Patients With First-Episode Schizophrenia Treated With Antipsychotics

**DOI:** 10.3389/fpsyt.2021.735623

**Published:** 2021-11-30

**Authors:** Ping Yin, Chao Zhao, Yang Li, Xiaoyi Liu, Lei Chen, Nan Hong

**Affiliations:** ^1^Department of Radiology, Peking University People's Hospital, Beijing, China; ^2^Department of Interventional Radiology, The First Hospital of Shanxi Medical University, Taiyuan, China

**Keywords:** schizophrenia, first-episode, antipsychotics, neuroimaging, functional magnetic resonance imaging

## Abstract

**Purpose:** Comprehensive and longitudinal brain analysis is of great significance for understanding the pathological changes of antipsychotic drug treatment in patients with schizophrenia. This study aimed to investigate the changes of structure, function, and network properties in patients with first-episode schizophrenia (FES) after antipsychotic therapy and their relationship with clinical symptoms.

**Materials and Methods:** A total of 30 patients diagnosed with FES and 30 healthy subjects matched for sex and age were enrolled in our study. Patients at baseline were labeled as antipsychotic-naive first-episode schizophrenia (AN-FES), and patients after antipsychotic treatment were labeled as antipsychotic treatment first-episode schizophrenia (AT-FES). The severity of illness was measured by using the PANSS and CGI score. Structural and functional MRI data were also performed. Differences in GMV, ALFF, and ReHo between the FES group and healthy control group were tested using a voxel-wise two-sample *t*-test, and the comparison of AN-FES group and AT-FES group was evaluated by paired-sample *t*-test.

**Results:** After the 1-year follow-up, the FES patients showed increased GMV in the right cerebellum, right inferior temporal gyrus, left middle frontal gyrus, parahippocampal gyrus, bilateral inferior parietal lobule, and reduced GMV in the left occipital lobe, gyrus rectus, right orbital frontal cortex. The patients also showed increased ALFF in the medial superior frontal gyrus and right precentral gyrus. For network properties, the patients showed reduced characteristic path length and increased global efficiency. The GMV of the right inferior parietal lobule was negatively correlated with the clinical symptoms.

**Conclusions:** Our study showed that the antipsychotic treatment contributed to the structural alteration and functional improvement, and the GMV alteration may be associated with the improvement of clinical symptoms.

## Introduction

Schizophrenia is a complex disease of the central nervous system, and a great deal of health care resources are devoted to these patients (1, 2). The genetic and environmental factors play an important role in the occurrence and development of schizophrenia. However, its pathogenic mechanism has not been fully defined, and further research is still needed. Functional magnetic resonance imaging (fMRI) has been widely used in the study of schizophrenia (3). The voxel based morphometry analysis is a common method to evaluate the change of gray matter volume (GMV). Resting-state fMRI is more stable and relatively easier to perform than task state fMRI, and is mainly used for brain functional connectivity and brain network analysis. Previous studies have shown structural and functional deficits in patients with schizophrenia, which may provide useful information for understanding the pathophysiology of schizophrenia (4).

Typical antipsychotics control symptoms by reducing the function of dopaminergic neurons (5). In addition to disease progression, many studies have confirmed that antipsychotics may affect the structure, function, and network characteristics of patients with schizophrenia to a certain extent (6, 7). However, previous studies have used a relatively single brain analysis method for cross-sectional design, which may not provide a comprehensive understanding of the effects of antipsychotics on patients with schizophrenia. Longitudinal and comprehensive studies on the effects of antipsychotic medication are necessary.

Therefore, in this study, we aimed to analyze the changes in the structure, function, and network properties of FES after antipsychotic therapy and their correlation with clinical symptoms.

## Materials and Methods

### Subjects

A total of 30 subjects diagnosed with FES were enrolled in our study. The FES patients were defined according to the patient edition of the Structured Clinical Interview for DSM-IV Axis I Disorders (SCID-IV) (8). Patients at baseline were labeled as antipsychotic-naive first-episode schizophrenia (AN-FES), and patients after antipsychotic treatment were labeled as antipsychotic treatment first-episode schizophrenia (AT-FES). Patients were selected from our hospital. The exclusion criteria for all subjects were a history of head injury, organic mental disorder, neurological disorder, serious medical or surgical illness, and a history of substance abuse. The healthy control subjects were recruited from the local community by advertisement. They had no history of psychiatric illness, as determined by the non-patient edition of the SCID, and no history of psychotic disorders among their first-degree relatives. This study was approved by our ethics committee, and all subjects provided fully informed written consent to participate in the study.

### Treatment and Response Assessment

The severity of illness was measured by senior clinical psychiatrists from the Department of Psychiatry using the Positive and Negative Syndrome Scale (PANSS) (9) and Clinical Global Impression Scales (CGI). CGI includes CGI for severity (CGI-S) and CGI for improvement (CGI-I). Patients received treatment according to the severity of the condition at baseline. During the follow-up, all patients received second generation antipsychotic drugs, including risperidone, aripiprazole and olanzapine. The PANSS and CGI score were assessed again after 1-year follow-up.

### Image Acquisition and Preprocessing

MRI data acquisition was performed on a 3.0T scanner (Signa HDx; GE Medical Systems, Milwaukee, WI, USA) equipped with an 8-channel phased-array brain coil at our hospital. Details regarding the image acquisition protocol and fMRI data preprocessing were presented in the Supplemental Material.

### GMV Calculation

The VBM8 toolbox (http://dbm.neuro.uni-jena.de/vbm.html) was used to estimate GMV. Structural MRI images were segmented into GM, WM, and cerebrospinal fluid using the standard segmentation model. After initial affine registration of the GM concentration map into the Montreal Neurological Institute (MNI) coordinate space, the GM concentration images were non-linearly warped using the DARTEL technique and then resampled to a voxel size of 1.5 × 1.5 × 1.5 mm. The GMV was obtained by multiplying the GM concentration map by the non-linear determinants that were derived from the spatial normalization step. Finally, the GMV images were smoothed using a Gaussian kernel of 6 × 6 × 6 mm full width at half maximum (FWHM).

### Amplitude of Low-Frequency Fluctuation (ALFF) and Regional Homogeneity (ReHo) Calculation

ALFF and ReHo were calculated using REST software (http://restfmri.net/forum/rest_v18). For standardization purposes, the ALFF of each voxel were divided by the global mean ALFF value within the whole-brain mask. Prior to the calculation of ReHo, the data were bandpass filtered (0.01–0.08 Hz). Subsequently, ReHo was determined on a voxel-by-voxel basis by calculating Kendall's coefficient of concordance of the time courses of a given voxel with those of its nearest 26 neighbors. Similar to the ALFF analysis, the ReHo of each voxel was divided by the global mean ReHo value.

### Construction of the Functional Brain Network and Properties Analysis

The software Gretna (http://www.nitrc.org/projects/gretna, v1.2.1) was used to reconstruct the functional brain network. The brain was divided into 90 regions by anatomical automatic labeling, and each region was regarded as a node of network. The pearson correlation coefficient between different nodes was calculated and a matrix with 90 × 90 symmetric distribution was established. Then the matrix was transformed into binary connection matrix, and the sparsity of connection matrix was regarded as a set threshold to construct brain functional networks. The whole and local topology parameters were analyzed at each sparsity. The whole topology parameters include small-world parameters and network efficiency. The small-world parameters include clustering coefficient (*C*_*p*_), characteristic path length (*L*_*p*_), normalized clustering coefficient (γ), normalized shortest path length (λ), and small-world index (σ). The network efficiency includes local efficiency (*E*_*loc*_) and global efficiency (*E*_*glob*_). The local topology parameters include knodal, Enodal, Degree centrality, and Betweenness centrality.

### Statistical Analysis

For voxel-based analyses of GMV, ALFF, and ReHo, the parametric test in the SPM8 package was used to test for statistical significance. Statistical inferences were determined with a voxel-level statistical threshold (*P* < 0.001, uncorrected). Differences in GMV, ALFF, and ReHo between the FES group and healthy control group were tested using a voxel-wise two-sample *t*-test, and the comparison of AN-FES group and AT-FES group was evaluated by paired-sample *t*-test.

The network parameters were analyzed by the software Gretna, and took the Sparsity as threshold, the range was 0.05 ≤ S ≤ 0.5, the interval value is 0.05, so the network analysis within this scope. In order to clarify the differences among subjects of the topology properties, the FES and healthy control group was tested by two-sample *t*-test, AN-FES group and AT-FES group was evaluated by paired-sample *t*-test. Meanwhile, the edge index of the network was considered qualitatively by using NBS, the threshold was *P* < 0.05, and the result was presented by BrainNet Viewer software.

Correlations among structural, functional abnormalities, global network properties, and severity of illness in AN-FES and AT-FES group were evaluated by partial correlation analysis. The gender and age were regarded as covariate for subsequent analysis. The correlation between the GMV, ALFF, ReHo, and global network properties in different regions in AT-FES group compared with AN-FES group and the difference value of positive, negative, general psychopathology, total scores on the PANSS, and CGI-S score before and after treatment and CGI-I score after treatment was analyzed. The threshold was *P* < 0.05.

## Results

### Demographic and Clinical Characteristics

Table 1 shows the demographic and clinical characteristics of the FES group and healthy control group. The CGI-I scores of the AN-FES group and the AT-FES group were 0 and 1.6 ± 0.7, respectively, indicating a significant therapeutic effect. The scores of CGI and PANSS in AT-FES group were lower than those in AN-FES group, and the difference was statistically significant (Table 2, *P* < 0.05).

**Table 1:** Demographic and characteristics of schizophrenia patients and healthy controls.

**Characteristics**	**AN-FES** **(*n* = 30)**	**Healthy controls** **(*n* = 30)**	***t/χ^2^*** **values**	***P*** **-value**
Age (years)	19.8 ± 3.1	20.8 ± 2.7	−1.3	0.202^a^
Gender (female/male)	17/13	17/13	1.0	0^b^
Clinical course (month)	8.5 ± 8.1	N/A	N/A	N/A
Age at onset (years)	19.5 ± 3.5	N/A	N/A	N/A

*The data are presented as the mean values ± standard deviations. NA, not applicable.*

a *A two-sample t-test was used to compare the differences in age across the groups.*

b *A chi-squared test was used to test the difference in gender across the groups*.

**Table 2:** The comparison of CGI and PANSS between AN-FES group and AT-FES group.

**Characteristics**	**AN-FES**	**AT-FES**	***t-*** **values**	***P*** **-value**
CGI-S	5.4 ± 0.8	1.8 ± 0.9	16.3	<0.001
CGI-I	0	1.6 ± 0.7	N/A	N/A
PANSS				
Positive score	21.8 ± 4.6	7.8 ± 2.7	11.2	<0.001
Negative score	18.8 ± 6.7	12.1 ± 5.7	5.4	<0.001
General score	37.7 ± 7.2	19.7 ± 4.2	13.1	<0.001
Total score	78.3 ± 14.6	40.5 ± 9.4	15.4	<0.001

*The data are presented as the mean values ± standard deviations. CGI-S, Clinical Global Impression Scales for severity; CGI-I, Clinical Global Impression Scales for improvement; PANSS, Positive and Negative Syndrome Scale; AN-FES, antipsychotic-naive first-episode schizophrenia; AT-FES, antipsychotic treatment first-episode schizophrenia; NA, not applicable*.

### Baseline Analysis of GMV, ALFF, and ReHo

Compared with healthy control group, the AN-FES group showed reduced GMV in the midbrain and left superior temporal gyrus (Supplementary Table 1, *P* < 0.001).

ALFF in the medial superior frontal gyrus, left middle frontal gyrus, right superior frontal gyrus, and right insula were all decreased in AN-FES group compared with healthy controls (Supplementary Table 1, *P* < 0.001).

In addition, AN-FES group also showed reduced ReHo in right superior frontal gyrus and right middle frontal gyrus and increased ReHo in the posterior cingulate cortex (Supplementary Table 1, *P* < 0.001).

### Baseline Analysis of Global and Local Network Properties

As 0.05 ≤ Sparsity ≤ 0.5, both the AN-FES group and healthy control group had higher γ value (all larger than 1) and approximately equal λ value. The σ > 1 of the two groups indicated that the two groups exhibited no difference in the small-world architecture of the functional brain network (*P* > 0.05). Compared with healthy controls, the C_p_, E_glob_, and E_loc_ of AN-FES were lower, and the L_p_ was higher, with statistically significant differences (Supplementary Table 2, *P* < 0.05).

There were significant differences in the betweenness centrality and degree centrality between AN-FES and the healthy control group, as shown in Supplementary Table 3.

### Longitudinal Analysis of GMV, ALFF, and ReHo

**Figure 1:**
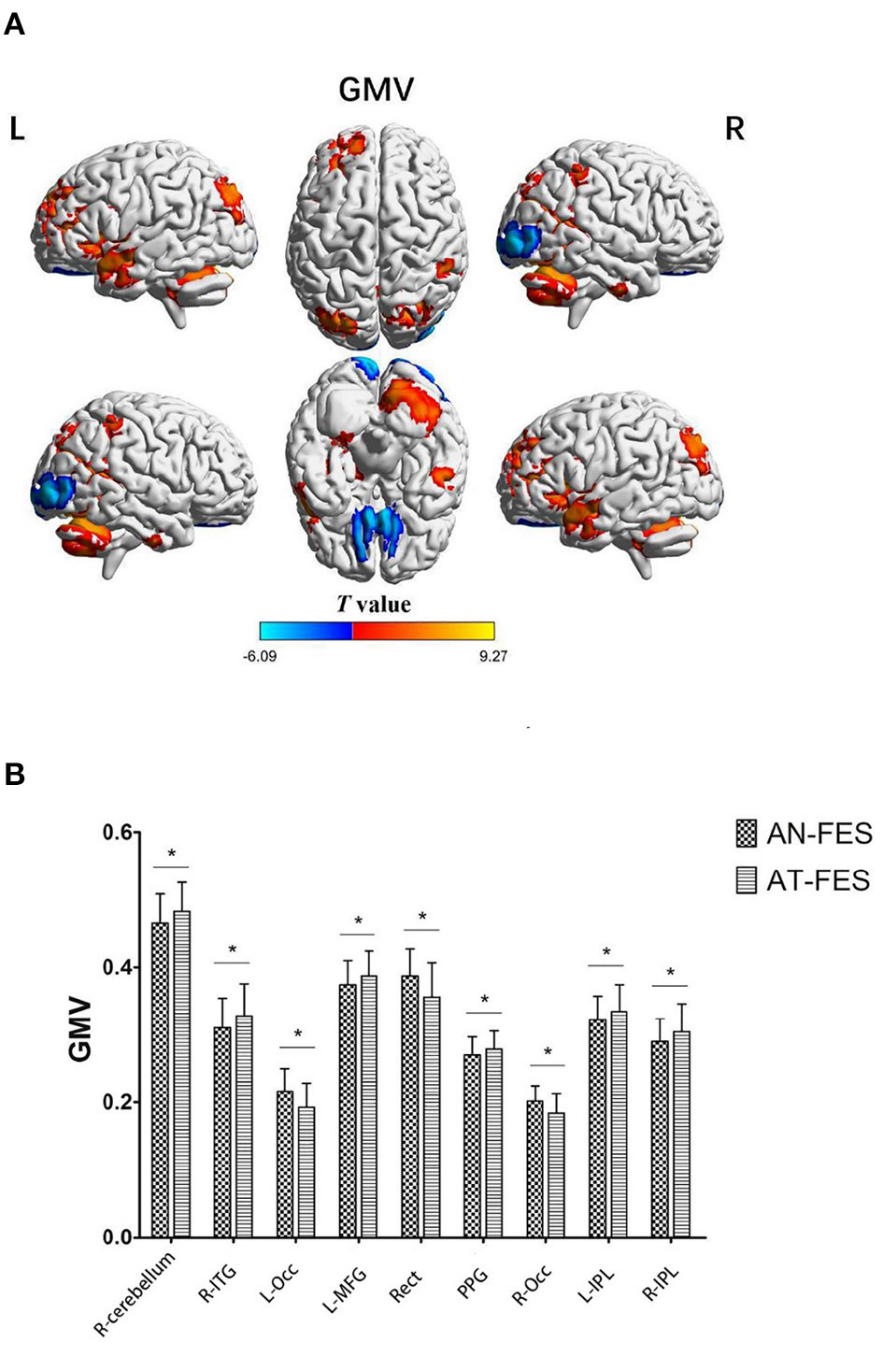
GMV differences between AN-FES group and AT-FES group. VBM analysis shows gray matter regions with GMV differences (*P* < 0.001, uncorrected) across the two groups **(A)**. A *post-hoc* ROI analysis shows the comparisons (*P* < 0.001, uncorrected) **(B)**. Error bars indicate the standard error of the mean. *P < 0.001, uncorrected. AN-FES, antipsychotic-naive first-episode schizophrenia; AT-FES, antipsychotic treatment first-episode schizophrenia; GMV, gray matter volume; L, left; R, right; ITG, inferior temporal gyrus; Occ, occipital lobe; MFG, middle frontal gyrus; Rect, gyrus rectus; PPG, parahippocampal gyrus; IPL, inferior parietal lobule.

The AT-FES group showed increased GM volume in the right cerebellum, right inferior temporal gyrus (ITG), left middle frontal gyrus (MFG), parahippocampal gyrus, bilateral IPL, and reduced GMV in the left occipital lobe, gyrus rectus, right orbital frontal cortex with that of the AN-FES group (Supplementary Table 4, Figure 1, *P* < 0.001).

**Figure 2:**
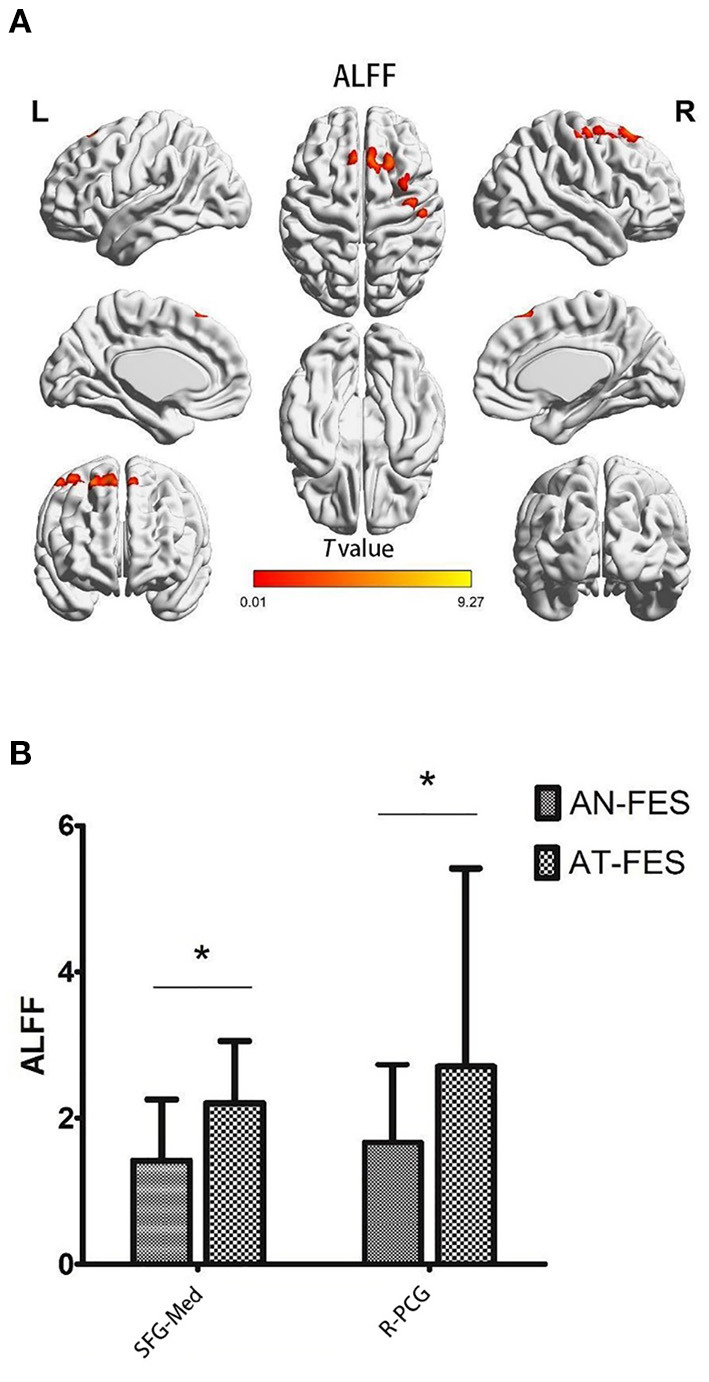
ALFF differences between AN-FES group and AT-FES group. Voxel-based analysis shows gray matter regions with ALFF differences (*P* < 0.001, uncorrected) across the two groups **(A)**. A *post hoc* ROI analysis shows the comparisons (*P* < 0.001, uncorrected) **(B)**. Error bars indicate the standard error of the mean. *P < 0.001, uncorrected. ALFF, amplitude of low frequency fluctuations; AN-FES, antipsychotic-naive first-episode schizophrenia; AT-FES, antipsychotic treatment first-episode schizophrenia; SFG, superior frontal gyrus; PCG, precentral gyrus; R, right; Med, medial.

The AT-FES patients showed increased ALFF in the medial superior frontal gyrus and right precentral gyrus (Supplementary Table 4, Figure 2, *P* < 0.001).

The ReHo in AT-FES group and AN-FES group showed no difference.

### Longitudinal Analysis of Global and Local Network Properties

**Figure 3:**
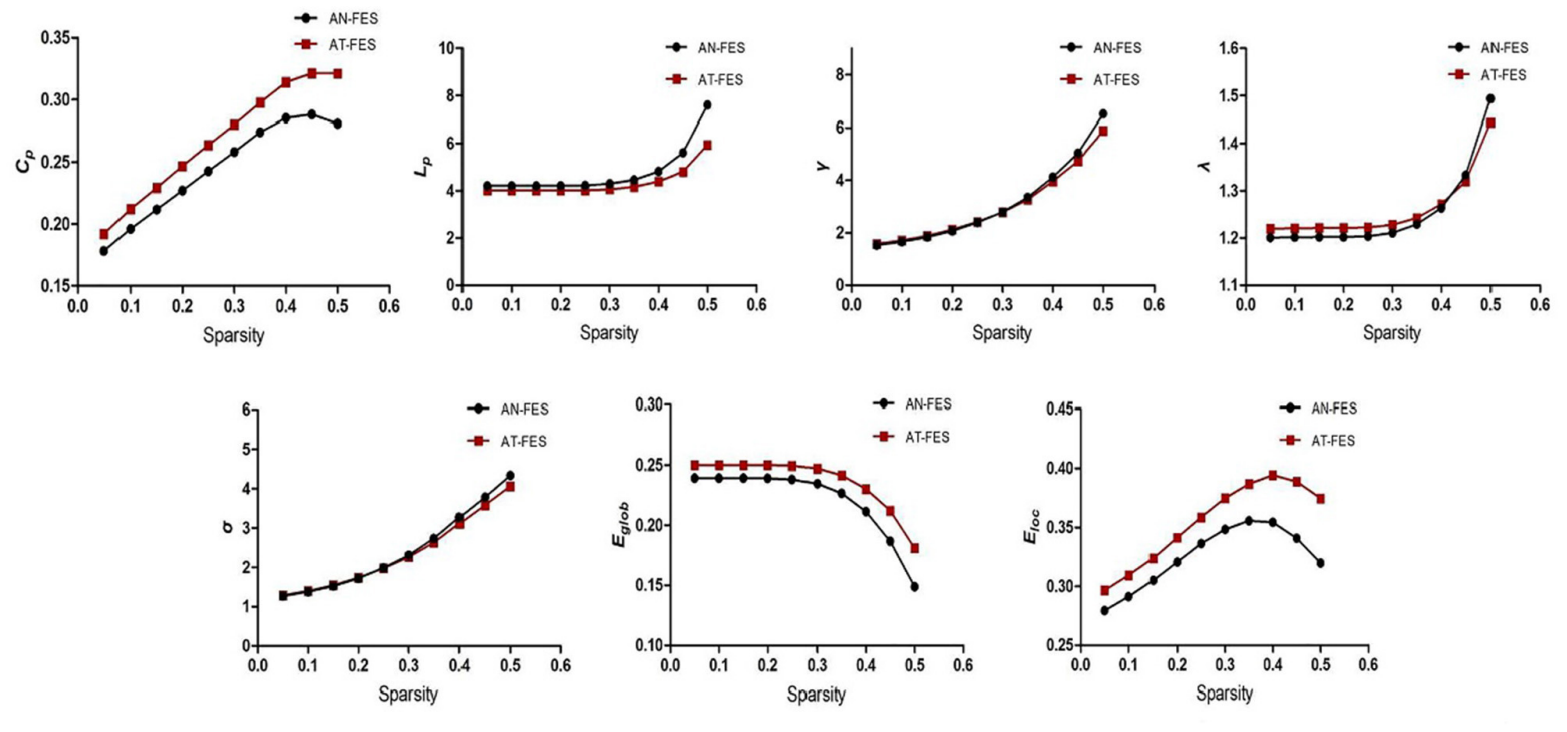
Comparison of the whole brain attributes between AN-FES group and AT-FES group at different sparsity. AN-FES, antipsychotic-naive first-episode schizophrenia; AT-FES, antipsychotic treatment first-episode schizophrenia.

As 0.05 ≤ Sparsity ≤ 0.5, both the AN-FES group and AT-FES group had higher γ value (all larger than 1) and approximately equal λ value. The σ > 1 in both groups indicated that the two groups showed no difference in the small-world structure of functional brain networks (Supplementary Table 5, Figure 3, *P* > 0.05). Compared with the AN-FES group, a lower L_p_ and higher E_glob_ were found in the AT-FES group (Supplementary Table 5, Figure 3, *P* < 0.05).

Multiple brain regions showed significant differences in the betweenness centrality and degree centrality between the AT-FES and AN-FES groups, details were shown in Supplementary Table 6.

**Figure 4:**
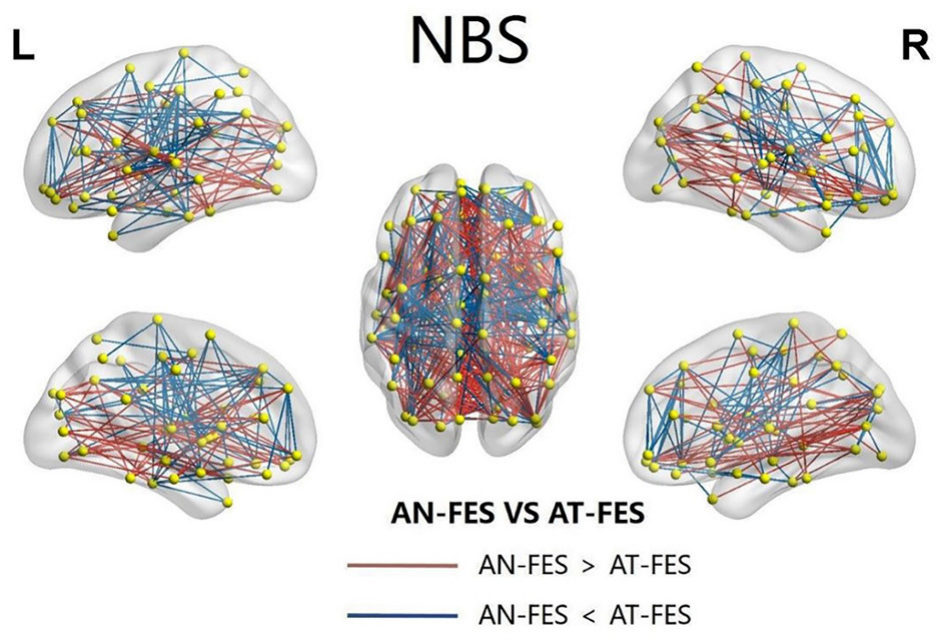
The abnormal NBS functional connectivity in AN-FES group and AT-FES group. NBS, network-based statistic; AN-FES, antipsychotic-naive first-episode schizophrenia; AT-FES, antipsychotic treatment first-episode schizophrenia.

The NBS showed the coexisting increased and reduced edge index of the network in AT-FES group and AN-FES group (Figure 4).

### The Correlation Analysis of Structural, Functional Abnormalities, Global Network Properties, and Severity of Illness

Partial correlation analysis showed that GMV of the left parietal lobe was positively correlated with the general psychopathological differences of PANSS before and after treatment (*r* = 0.519, *P* = 0.016). The details were shown in Supplementary Table 7.

## Discussion

In this study, we analyzed the differences in brain structure, function, and network topological characteristics between the FES group and healthy controls at baseline, and explored the effect of antipsychotics on FES after 1 year of follow-up. Multiple brain regions showed reduced GMV, ALFF, and ReHo at baseline, and the GMV of FES after treatment showed coexisting reduced and increased brain regions. The partial correlation analysis showed the correlation of GMV, network topological properties, and clinical symptoms. Our study provides comprehensive and valuable longitudinal information on FES.

MRI confirmed a decrease in GMV and white matter volume (WMV), with some investigators noting that the decrease in GMV (2%) was more pronounced than the decrease in WMV (1%) (10, 11). Thus, the study of GMV in schizophrenia patients may helpful for pathogenesis and pathologic changes research. Our results showed reduced GMV in the midbrain and left superior temporal gyrus at baseline. Most previous studies indicated the reduction of GMV usually existed in the prefrontal cortex, temporal lobe and parietal cortex (12–14). Nopoulos et al. (15) also found that the schizophrenia patients had significantly smaller midbrain measures compared with control subjects. Since dopamine disorders are central to the pathophysiology of psychosis, the midbrain contains the extranuclear body of all dopamine neurons in the human brain.

In our study, ALFF and ReHo in some brain regions decreased at baseline, suggesting impaired local brain activity and functional connectivity in FES. The schizophrenia patients showed obvious ALFF reduction in bilateral occipital lobe, motorsensory cortex, and posterior parietal cortex by comparison with healthy subject (16). Some studies pointed out that the FES had reduced ALFF in prefrontal lobe, and this change was more obvious in early stage of the disease (17, 18). The increased or reduced ReHo all indicated the abnormity of synchrony of spontaneous neuron activity and coordination mechanism, and evidence abounds that the ReHo abnormity existed in prefrontal lobe, superior temporal gyrus, and posterior cingulate cortex (19–21). Salvador first used the automated anatomical labeling as a template for brain region segmentation and built a whole brain network in 2005 (22). Some researchers (23–25) indicated the *C*_*p*_, *E*_*glob*_, *E*_*loc*_, and node degree were reduced and *L*_*p*_ was increased in schizophrenia patients, which was consistent with our research results.

Our study showed coexisting reduced and increased brain regions in AT-FES patients, and the increased GMV of the left parietal lobule was negatively correlated with clinical symptoms, indicating that the change of GMV after treatment was correlated with improvement of schizophrenia. The antipsychotics may influence the normal physiological process of brain and the progress of schizophrenia, and then have an effect on GMV change of brain (12). Most studies showed the abnormity of GMV exist mainly in temporal lobe, parietal lobe and occipital lobe, and this change was correlated with positive symptoms of schizophrenia, cognitive, and emotional function impediment (26, 27). In our study, the AT-FES group showed reduced GMV in the left occipital lobe, gyrus rectus, right orbital frontal cortex with that of the AN-FES group. The gyrus rectus, right orbital frontal cortex belong to the prefrontal cortex, which is thought to be an important brain region for processing emotions and cognition, such as working memory, and is associated with self-awareness. The present study found that the occipital lobe GMV decreased in the FES group, which was consistent with the conclusion of the previous study (28). Some studies found that the total dose of antipsychotics may predict the GMV change in FES (29, 30). A recent study identified the relationship between antipsychotics and structural changes and suggested that GMV may have the potential to predict antipsychotic response in patients with FES (31). However, due to the small sample size and other factors, further researches are needed in the future.

Our study showed the increased ALFF in the medial superior frontal gyrus and the right precentral gyrus of FES after treatment. Lui et al. reported the ALFF was increased after treatment in some brain regions such as the prefrontal lobe and parietal lobe (17). The medial superior frontal gyrus participates in task state cognition and motor behavior, and also was a part of the default network. In this study, ALFF in the medial superior frontal gyrus decreased at baseline and increased after treatment, suggesting that antipsychotics may have an impact on the local function of this brain region. The anterior central gyrus is involved in the processing of cognitive information related to motor function, and cognitive abnormalities and intentional control disorders may occur after injury. A previous study showed that antipsychotic drugs ameliorate abnormalities in the anterior central gyrus (32).

There are few studies on the effect of antipsychotics on network properties in patients with schizophrenia. A study indicated that the antipsychotics may improve the reduced *E*_*glob*_ and increased *C*_*p*_ (33). In our study, the brain regions with different node properties before and after treatment in FES were the auditory network, which mainly included the insular lobe, transverse gyrus and supramarginal gyrus; the subcortical network mainly included the amygdala, putamen and pallidum; and the sensorimotor network mainly included the pre-central gyrus and post-central gyrus. Most of the brain regions that reduced betweenness centrality at baseline improved after treatment, and this change was associated with remission of clinical symptoms. Previous studies have also shown the effectiveness of antipsychotics in improving auditory hallucinations (34).

Our study has several limitations. First, the results may be limited by the small sample size. Second, due to the exploratory nature of the study, we used uncorrected instead of corrected, as to not inhibit hypothesis generating research by overcorrection for false discovery (35). Third, a prospective, within-subject, counterbalanced drug/placebo design would be preferable to the naturalistic design of the present study, but such a study would not be feasible or ethically justified (36). However, our study is still meaningful and may provide relatively more useful information for longitudinal design and overall analysis.

In conclusion, our study showed changes in the structure, function, and network properties in FES after the antipsychotic treatment and their correlation with clinical symptoms. Our study may help to provide a more complete understanding of the pathological changes in patients with schizophrenia following antipsychotic medication.

## Data Availability Statement

The original contributions presented in the study are included in the article/Supplementary Material, further inquiries can be directed to the corresponding author.

## Ethics Statement

The study was approved by the Ethics Committee of Peking University People's Hospital. Written informed consent to participate in this study was provided by the participants' legal guardian/next of kin.

## Author Contributions

NH: guarantor of integrity of the entire study and manuscript review. PY and NH: study concepts. PY and CZ: study design, definition of intellectual content, clinical studies, and manuscript editing. PY and YL: literature research and data acquisition. PY and XL: experimental studies and data analysis. PY: statistical analysis. PY, LC, and YL: manuscript preparation. All authors contributed to the article and approved the submitted version.

## Conflict of Interest

The authors declare that the research was conducted in the absence of any commercial or financial relationships that could be construed as a potential conflict of interest.

## Publisher's Note

All claims expressed in this article are solely those of the authors and do not necessarily represent those of their affiliated organizations, or those of the publisher, the editors and the reviewers. Any product that may be evaluated in this article, or claim that may be made by its manufacturer, is not guaranteed or endorsed by the publisher.
