# Supplementary material for: Changes in Brain Structure, Function, and Network Properties in Patients With First-Episode Schizophrenia Treated With Antipsychotics
Source: Front Psychiatry. 2021 Nov 30;12:735623. doi: 10.3389/fpsyt.2021.735623 (PMC8668948; doi:10.3389/fpsyt.2021.735623)
Supplement: Supplementary file 1 [file Data_Sheet_1.docx]

**Supplementary Materials**

**Text**

***S1.*** *MRI data acquisition and preprocessing*

MRI data acquisition was performed on a 3.0 T scanner (Signa HDx; GE Medical Systems, Milwaukee, WI, USA) equipped with an 8-channel phased-array brain coil at the People's Hospital, Peking University, China. The patients had not taken any medicine for 24 h before the scan and were quietly lying awake in the scanner with their eyes closed. Foam pads and earplugs were used to minimize head motion and scanner noise. T1-weighted imaging was achieved for GM volume analysis using three-dimensional fast spoiled gradient-recalled acquisition in the steady state (3D-FSPGR) in 166 coronal slices (acquisition matrix = 256 × 256; echo time = 3.9 ms; repetition time = 9.6 ms; field of view = 240 × 240 mm; slice thickness/gap = 1.2 /0 mm; scanning time = 3 min 14 s). The functional images were obtained using an echo-planar imaging sequence with the following parameters to minimize the potential motion artifacts: thickness/gap = 5 /1.2 mm, repetition time = 2000 ms, echo time = 40 ms, flip angle = 90°, field of view = 240 × 240 mm^2^, matrix = 64 × 64, and number of excitations = 122 slices. The scan lasted for 4 min.

In Matlab2011 platform environment, all the resting-state fMRI data preprocessing was carried out with statistical parametric mapping (SPM8, [http://www.fil.ion.ucl.ac.uk/spm/](file:///C:/Users/alex/Downloads/Introduction20150320最新_pu.docx) and Gretna, <http://www.nitrc.org/projects/gretna>, v1.2.1). The data were preprocessed using the following steps. The first 5 volumes from each participant were discarded to allow the signal to reach equilibrium and to allow the participant to adapt to the scanning noise. The remaining 115 volumes were corrected for the acquisition time delay between slices. Realignment was then performed to correct the motion displacement. All participants’ data were within the defined motion thresholds (translational or rotational motion parameters < 2 mm or 2°). We calculated framewise displacement (FD), which indexes the volume‑to‑volume changes in head position. Several nuisance covariates were regressed out from the motion-corrected fMRI data, including six motion parameters and their first-order derivatives, the global brain signal, the white matter signal, the cerebrospinal fluid signal, and spike volumes with FD higher than 0.5. The fMRI images were further spatially normalized to the Montreal Neurological Institute (MNI) echo-planar imaging template and resampled to 3 mm cubic voxels. Data were smoothed with a 6 mm FWHM kernel to confirm the Gaussian field model.

**Tables**

**Table S1**

Brain areas with significant differences in GMV, ALFF and ReHo across the AN-FES group and healthy control group

| Brain region | Brodmann areas | Cluster size (voxels) | Peak *t* values | Coordinates in MNI | | |
| --- | --- | --- | --- | --- | --- | --- |
|  |  |  |  | X | Y | Z |
| GMV |  |  |  |  | | |
| midbrain | — | 187.0 | 6.8 | -9.0 | -24.0 | -3.0 |
| L-STG | 22.0 | 381.0 | 6.1 | -39.0 | 12.0 | -15.0 |
| ALFF |  |  |  |  |  |  |
| SFG_Med | 10.0 | 155.0 | 6.2 | 21.0 | 66.0 | 24.0 |
| L-MFG | 8.0 | 194.0 | 5.4 | -27.0 | 33.0 | 51.0 |
| R-SFG | 6.0 | 203.0 | 5.5 | 21.0 | 6.0 | 66.0 |
| R-insula | 13.0 | 54.0 | 5.2 | 33.0 | 15.0 | 9.0 |
| ReHo |  |  |  |  |  |  |
| R-SFG | 8.0 | 48.0 | 5.1 | 39.0 | 36.0 | 45.0 |
| R-MFG | 10.0 | 113.0 | 5.1 | 42.0 | 48.0 | 30.0 |
| PCC | 29.0 | 54.0 | -4.3 | -9.0 | -45.0 | 18.0 |

Abbreviations: AN-FES, antipsychotic-naive first-episode schizophrenia; GMV, gray matter volume; ALFF, amplitude of low frequency fluctuations; ReHo, regional homogeneity; STG, superior temporal gyrus; SFG, superior frontal gyrus; MFG, middle frontal gyrus; PCC, posterior cingulate cortex; L, left; Med, medial; R, right.

**Table S2**

Differences in global properties of brain networks across the AN-FES group and healthy control group

| Parameters | AN-FES | Helthy controls | *t* values | *P* value |
| --- | --- | --- | --- | --- |
| *C_p_* | 0.111±0.020 | 0.126±0.014 | 3.372 | < 0.001 |
| *L_p_* | 2.099±0.297 | 1.876±0.123 | -3.802 | < 0.001 |
| *γ* | 1.365±0.246 | 1.360±0.170 | -0.090 | 0.929 |
| *λ* | 0.560±0.020 | 0.568±0.016 | 1.782 | 0.080 |
| *σ* | 1.077±0.200 | 1.065±0.140 | -0.285 | 0.778 |
| *E_glob_* | 0.100±0.012 | 0.109±0.007 | 3.511 | < 0.001 |
| *E_loc_* | 0.148±0.024 | 0.166±0.014 | 3.686 | < 0.001 |

Notes: AN-FES, antipsychotic-naive first-episode schizophrenia; *C_p_*, clustering coefficient; *L_p_*, characteristic path length;*γ*, standardized clustering coefficient;*λ*, standardized characteristic path length; *σ*, small-worldness; *E_glob_*, global properties; *E_loc_*, local properties.

**Table S3**

Differences in local properties of brain networks across the AN-FES group and healthy control group

| Brain region | AN-FES | Helthy controls | *P* value |
| --- | --- | --- | --- |
| Degree centrality |  |  |  |
| L-SFG | 2.941±1.383 | 3.916±1.104 | 0.004 |
| R-SFG | 2.919±1.315 | 3.783±1.055 | 0.007 |
| R-Orb | 2.941±1.535 | 3.690±1.320 | 0.050 |
| L-IFG | 3.379±0.944 | 4.088±1.271 | 0.018 |
| L-insula | 3.826±1.658 | 4.830±1.643 | 0.023 |
| R-insula | 3.769±1.696 | 4.681±1.583 | 0.037 |
| R-amygdala | 3.160±1.240 | 3.929±1.564 | 0.040 |
| L-PCG  Pcu | 3.177±1.549 | 4.217±1.758 | 0.019 |
|  | 2.583±1.012 | 3.202±0.957 | 0.019 |
| L-Cau | 3.160±1.250 | 3.844±1.318 | 0.046 |
| R-Cau | 2.861±1.392 | 3.546±1.222 | 0.049 |
| L-putamen | 3.644±1.347 | 4.686±1.376 | 0.005 |
| R-putamen | 3.283±1.514 | 4.531±1.643 | 0.004 |
| R-pallidum  L-GTT | 3.033±0.783 | 3.033±0.783 | 0.002 |
|  | 3.501±1.432 | 4.392±1.554 | 0.026 |
| betweenness centrality |  |  |  |
| R-SFG | 39.989±27.073 | 23.524±17.645 | 0.008 |
| R-cingulate gyrus | 29.493±24.619 | 49.841±31.199 | 0.007 |
| R-Hippo | 35.046±24.535 | 18.279±11.259 | 0.001 |
| R-calcareous gyrus | 39.658±28.614 | 19.106±22.005 | 0.003 |
| R-lingual gyrus | 35.623±32.577 | 18.623±18.866 | 0.018 |
| R-PCG | 34.525±23.913 | 21.728±21.028 | 0.033 |
| L-SPL | 22.290±16.520 | 42.958±32.697 | 0.003 |
| R-paracentral lobule | 32.862±22.874 | 19.906±21.267 | 0.028 |
| R-MTG | 40.465±41.320 | 21.938±16.688 | 0.029 |
| R-ITG | 19.099±17.848 | 31.121±24.456 | 0.035 |

Abbreviations: AN-FES, antipsychotic-naive first-episode schizophrenia; SFG, superior frontal gyrus; Orb, orbital frontal cortex; IFG, inferior frontal gyrus; PCG, posterior central gyrus; Pcu, precuneus; Cau, caudate; GTT, gyri temporales transversi; Hippo, hippocampus; PCG, posterior central gyrus; SPL, superior parietal lobule; MTG, middle temporal gyrus; ITG, inferior temporal gyrus; L, left; R, right.

**Table S4**

Brain areas with significant differences in GMV, ALFF and ReHo across the AN-FES group and AT-FES group

| Brain region | Brodmann areas | Cluster size (voxels) | Peak *t* values | Coordinates in MNI | | |
| --- | --- | --- | --- | --- | --- | --- |
|  |  |  |  | X | Y | Z |
| GMV |  |  |  |  | | |
| R-cerebellum | — | 10071 | -8.3 | 19.5 | -78.0 | -18.0 |
| R-ITG | 20 | 304 | -4.8 | 57.0 | -7.5 | -40.5 |
| L-Occ | 18 | 155 | 6.0 | 1.5 | -99.0 | -6.0 |
| L-MFG | 10 | 194 | -7.9 | -42.0 | -12.0 | -18.0 |
| Rect | 11 | 203 | 5.1 | 1.5 | 12.0 | -28.5 |
| PPG | 36 | 302 | -4.6 | -18.0 | -16.5 | -12.0 |
| R-Occ | 19 | 1415 | 5.8 | 42.0 | -93.0 | -1.5 |
| L-IPL | 40 | 799 | -6.6 | -34.5 | -79.5 | 28.5 |
| R-IPL | 41 | 391 | -4.3 | 54.0 | -40.5 | 55.5 |
| ALFF |  |  |  |  |  |  |
| SFG-Med | 10 | 71 | -5.0 | 9.0 | 27.0 | 60.0 |
| R-PCG | 4 | 35 | -4.7 | 39.0 | 9.0 | 63.0 |

Abbreviations: AN-FES, antipsychotic-naive first-episode schizophrenia; AT-FES, antipsychotic treatment first-episode schizophrenia; GMV, gray matter volume; ALFF, amplitude of low frequency fluctuations; ReHo, regional homogeneity; ITG, inferior temporal gyrus; Occ, occipital lobe; MFG, middle frontal gyrus; Rect, gyrus rectus; PPG, parahippocampal gyrus; IPL, inferior parietal lobule; SFG, superior frontal gyrus; PCG, precentral gyrus; L, left; R, right; Med, medial.

**Table S5**

Differences in global properties of brain networks across the AN-FES group and AT-FES group

| Parameters | AN-FES | AT-FES | *t* values | *P* value |
| --- | --- | --- | --- | --- |
| *C_p_* | 0.111±0.020 | 0.121±0.015 | -1.952 | 0.061 |
| *L_p_* | 2.099±0.297 | 1.924±0.210 | 2.411 | 0.022 |
| *γ* | 1.365±0.246 | 1.331±0.201 | 0.595 | 0.557 |
| *λ* | 0.560±0.020 | 0.564±0.014 | -0.878 | 0.387 |
| *σ* | 1.077±0.200 | 1.048±0.157 | 0.615 | 0.544 |
| *E_glob_* | 0.100±0.012 | 0.107±0.009 | -2.212 | 0.035 |
| *E_loc_* | 0.148±0.024 | 0.161±0.018 | -2.037 | 0.051 |

Notes: AN-FES, antipsychotic-naive first-episode schizophrenia; AT-FES, antipsychotic treatment first-episode schizophrenia; *C_p_*, clustering coefficient; *L_p_*, characteristic path length; *γ*, standardized clustering coefficient; *λ*, standardized characteristic path length; *σ*, small-worldness; *E_glob_*, global properties; *E_loc_*, local properties.

**Table S6**

Differences in local properties of brain networks across the AN-FES group and AT-FES group

| Brain region | AN-FES | AT-FES | *P* value |
| --- | --- | --- | --- |
| Degree centrality |  |  |  |
| L-SFG | 2.941±1.406 | 3.916±1.085 | 0.011 |
| R-SFG | 2.919±1.338 | 3.783±1.037 | 0.023 |
| R-IFG | 3.379±0.960 | 4.088±1.249 | 0.014 |
| L-insula | 3.826±1.686 | 4.830±1.615 | 0.038 |
| R-amygdala | 3.160±1.261 | 3.929±1.538 | 0.049 |
| L-PCG | 3.177±1.576 | 4.217±1.729 | 0.040 |
| L-Pcu | 2.583±1.030 | 3.202±0.941 | 0.030 |
| L-putamen | 3.644±1.370 | 4.686±1.353 | 0.016 |
| R-putamen | 3.283±1.540 | 4.531±1.615 | 0.013 |
| R-pallidum  L-GTT | 3.033±0.796 | 3.990±1.403 | 0.012 |
|  | 3.501±1.456 | 4.392±1.527 | 0.030 |
| betweenness centrality |  |  |  |
| R-precentral gyrus | 38.798±31.597 | 22.478±17.313 | 0.022 |
| R-supramarginal gyrus | 25.148±19.943 | 40.105±28.738 | 0.048 |

Abbreviations: AN-FES, antipsychotic-naive first-episode schizophrenia; AT-FES, antipsychotic treatment first-episode schizophrenia; SFG, superior frontal gyrus; IFG, inferior frontal gyrus; PCG, posterior central gyrus; Pcu, precuneus; Cau, caudate; GTT, gyri temporales transversi; L, left; Med, medial; R, right.

**Table S7**

Partial correlations of GMV, ALFF and network property with clinical symptoms in the AT-FES group.

|  | PANSS-Total | | PANSS-Positive | | | PANSS-Negative | |  |
| --- | --- | --- | --- | --- | --- | --- | --- | --- |
|  | *r* | *P* | | *r* | *P* | *r* | *P* | |
| GMV |  |  | |  |  |  |  | |
| R-cerebellum | 0.228 | 0.321 | | 0.353 | 0.117 | -0.107 | 0.644 | |
| R-ITG | -0.268 | 0.241 | | 0.071 | 0.761 | -0.405 | 0.068 | |
| L-MFG | 0.316 | 0.163 | | 0.393 | 0.078 | 0.038 | 0.870 | |
| PPG | -0.014 | 0.951 | | 0.417 | 0.060 | -0.193 | 0.402 | |
| L-IPL | 0.353 | 0.116 | | 0.409 | 0.066 | 0.315 | 0.164 | |
| R-IPL | 0.207 | 0.369 | | 0.181 | 0.433 | -0.215 | 0.349 | |
| L-Occ | -0.039 | 0.865 | | 0.045 | 0.846 | -0.089 | 0.703 | |
| Rect | 0.332 | 0.142 | | 0.203 | 0.377 | 0.096 | 0.678 | |
| R-Occ | 0.081 | 0.727 | | -0.011 | 0.964 | 0.177 | 0.443 | |
| ALFF |  |  | |  |  |  |  | |
| SFG-Med | 0.128 | 0.581 | | -0.191 | 0.406 | 0.268 | 0.241 | |
| R-PCG | -0.278 | 0.222 | | 0.249 | 0.276 | -0.170 | 0.462 | |
| Network property |  |  | |  |  |  |  | |
| *L_p_* | 0.341 | 0.131 | | -0.284 | 0.212 | 0.187 | 0.416 | |
| *E_glob_* | -0.234 | 0.306 | | 0.326 | 0.149 | -0.105 | 0.652 | |

|  | PANSS-General | | CGI-S | | | CGI-I | |  |
| --- | --- | --- | --- | --- | --- | --- | --- | --- |
|  | *r* | *P* | | *r* | *P* | *r* | *P* | |
| GMV |  |  | |  |  |  |  | |
| R-cerebellum | 0.179 | 0.438 | | 0.031 | 0.894 | -0.287 | 0.206 | |
| R-ITG | -0.322 | 0.155 | | -0.169 | 0.464 | 0.006 | 0.980 | |
| L-MFG | 0.265 | 0.245 | | 0.062 | 0.790 | -0.150 | 0.515 | |
| PPG | 0.022 | 0.924 | | -0.065 | 0.781 | -0.106 | 0.648 | |
| L-IPL | 0.519 | 0.016 | | 0.386 | 0.084 | -0.397 | 0.075 | |
| R-IPL | 0.030 | 0.898 | | 0.072 | 0.758 | 0.045 | 0.845 | |
| L-Occ | -0.123 | 0.597 | | -0.039 | 0.867 | -0.056 | 0.810 | |
| Rect | 0.301 | 0.185 | | 0.359 | 0.110 | -0.421 | 0.058 | |
| R-Occ | 0.171 | 0.458 | | 0.102 | 0.659 | -0.213 | 0.354 | |
| ALFF |  |  | |  |  |  |  | |
| SFG-Med | 0.109 | 0.639 | | 0.078 | 0.736 | 0.029 | 0.900 | |
| R-PCG | -0.071 | 0.761 | | 0.292 | 0.200 | -0.343 | 0.128 | |
| Network property |  |  | |  |  |  |  | |
| *L_p_* | 0.107 | 0.645 | | 0.065 | 0.780 | 0.090 | 0.698 | |
| *E_glob_* | 0.001 | 0.997 | | -0.040 | 0.865 | -0.111 | 0.633 | |

Abbreviations: AN-FES, antipsychotic-naive first-episode schizophrenia; AT-FES, antipsychotic treatment first-episode schizophrenia; ALFF, amplitude of low frequency fluctuations; Cau, caudate; GMV, gray matter volume; Hippo, hippocampus; IPL, inferior parietal lobule; L, left; Med, medial; MTG, middle temporal gyrus; Occ, occipital lobe; Orb, orbital frontal cortex; PANSS, Positive and Negative Syndrome Scale; CGI-S, Clinical Global Impression Scales for severity; CGI-I, Clinical Global Impression Scales for improvement; R, right; Rect, gyrus rectus; ReHo, regional homogeneity; SFG, superior frontal gyrus; SPL, superior parietal lobule; Pcu, precuneus. L, left. Med, medial. R, right.
